# Efficacy of artemisinin-based combination therapies and prevalence of molecular markers associated with artemisinin, piperaquine and sulfadoxine-pyrimethamine resistance in Sierra Leone

**DOI:** 10.1016/j.actatropica.2018.06.016

**Published:** 2018-09

**Authors:** Samuel J. Smith, Anitta R.Y. Kamara, Foday Sahr, Mohamed Samai, Alpha S. Swaray, Didier Menard, Marian Warsame

**Affiliations:** a National Malaria Control Programme, Sierra Leone; b College of Medicine and Allied Health Services, Sierra Leone; c National Laboratory, Connaught Teaching Hospital, Sierra Leone; d Malaria Genetic and Resistance Group, Biology of Host-Parasite Interactions Unit, Department of Parasites and Insect Vectors, Institut Pasteur, Paris, France; e Global Malaria Programme, World Health Organization, 20 Avenue Appia, 1211 Geneva 27, Switzerland

**Keywords:** Artemether–lumefantrine, Artesunate–amodiaquine, Dihydroartemisinin–piperaquine, *pfk13*, *pfplasmepsin2*, *pfdhfr/pfdhps*, Sierra Leone

## Abstract

Currently, the national malaria control programme (NMCP) of Sierra Leone recommends artesunate–amodiaquine (ASAQ) and artemether–lumefantrine (AL) as first- and second-line treatment for uncomplicated malaria, respectively, and artesunate + sulfadoxine–pyrimethamine (SP) for intermittent preventive treatment during pregnancy and for infants. In 2016, the NMCP conducted a study to assess the clinical and parasitological responses of children under five years to ASAQ, AL and dihydroartemisinin–piperaquine (DHA/PPQ) according to the WHO protocol. Day-0 samples were tested for mutations in the *Kelch 13* gene *(pfk13)* and dihydrofolate reductase/dihydropteroate synthase (*pfdhfr/pfdhps*) genes associated with artemisinin and SP resistance, respectively, and amplification in the *pfplasmepsin2* gene for piperaquine resistance. A total of 295 (ASAQ = 128, AL = 64 and DHA/PPQ = 103) eligible children were enrolled at three sites. PCR-corrected 100% adequate clinical and parasitological response and no parasitaemia on day-3 were observed for all patients in each treatment group. Of the 278 samples with interpretable molecular data, only 2.2% carried non-synonymous *pfk13* mutants (A578S, I646T), which are not associated with artemisinin resistance. None of the 103 day-0 samples from the DAH/PPQ group had *pfplasmepsin2* gene amplification, confirming the absence of piperaquine resistance. The prevalence of the triple *pfdhfr* mutant (N51I/C59R/S108N) was close to or reached fixation (97.4–100%). For combined *pfdhfr/pfdhps* mutation, 55–71% carried the quadruple (N51I/C59R/S108N+A437G) mutant and about 10% the quintuple mutant N51I/C59R/S108N+A437G/K540E. Our findings confirm that ASAQ, AL and DHA/PPQ were highly effective for the treatment of uncomplicated malaria in the study areas, and neither *pfk13* validated mutations nor *pfplasmepsin2* multiple copies were found. The very low prevalence of the quintuple mutant in this study supports the NMCP’s decision to introduce intermittent preventive treatment for infants with SP in the districts with high malaria transmission.

## Highlights

• The evaluated artemisinin combination therapies were highly efficacious for the treatment of uncomplicated malaria.
• *Pfk13* mutations and *Pfplasmepsin2* multiple copies, associated with artemisinin and piperaquine resistance, respectively, were absent.
• The very low prevalence of the quintuple mutant detected supports the introduction of intermittent preventive treatment for infants with SP.
• The efficacy and molecular markers of the recommended artemisinins and partners drugs should be monitored continuously.

## 1 Introduction

Effective antimalarial therapy is necessary to cure malaria infection, prevent progression to severe disease and save lives. To counteract the resistance of *Plasmodium falciparum* first to chloroquine and later to other monotherapies such as sulfadoxine–pyrimethamine (SP), WHO recommends artemisinin-based combination therapy (ACT) for the treatment of uncomplicated *P. falciparum* malaria (WHO, 2015). These drugs are artesunate–amodiaquine (ASAQ), artemether–lumefantrine (AL), artesunate–sulfadoxine–pyrimethamine (ASSP), dihydroartemisinin–piperaquine (DHA/PPQ) and artesunate–mefloquine (ASMQ). Furthermore, WHO recommends intermittent preventive treatment with SP in pregnancy (IPTp) and in infants (IPTi) living in settings with moderate-to-high malaria transmission in order to mitigate the adverse consequences of malaria infection in these risk groups (WHO, 2010a, 2014). According to the recommendation, pregnant women receive SP treatment at each antenatal visit until delivery, starting in the second trimester of pregnancy, with subsequent doses given at least 1 month apart. For infants, the therapeutic course of SP is delivered through the Expanded Programme on Immunization at defined intervals corresponding to routine vaccination schedules (10 weeks, 14 weeks and 9 months of age).

Resistance of *P. falciparum* to antimalarial drugs poses a serious threat to the fight against malaria. The threat is real in the Greater Mekong sub-region, where resistance to artemisinin has emerged independently and spread (Ashley et al., 2014; Takala-Harrison et al., 2015; Tun et al., 2015; Menard et al., 2016; WHO, 2017a). Recent studies show high treatment failure rates with DHA/PPQ as well as piperaquine and/or artemisinin resistance in several countries in the subregion (Leang et al., 2015; Thanh et al., 2017; Phuc et al., 2017; Imwong et al., 2017). These developments indicate the importance of vigilance in malaria-endemic countries for the possible emergence of resistance to artemisinin and partner drugs and treatment failures with ACT. The therapeutic efficacy study is the gold standard for monitoring the efficacy of antimalarial medicines in order to inform national malaria treatment policy. Monitoring of molecular markers for resistance to antimalarial drugs could support *in vivo* studies by confirming parasite resistance. Artemisinin resistance has been shown to be associated with point mutations in the *P. falciparum Kelch13* (*pf*k*13*) gene (Ariey et al., 2014; Straimer et al., 2015); and specific point polymorphisms that accumulate in several codons in *P. falciparum* dihydrofolate reductase (*pfdfhr*)/dihydropteroate synthase (*pfdhps*) genes are implicated in sulfadoxine and pyrimethamine resistance, respectively (Roper et al., 2003); and the presence of the quintuple mutant (*pfdhfr* triple N51I/C59R/S108N plus *pfdhps* double A437G/K540E) has been associated with SP treatment failure (Kublin et al., 2002; Mombo-Ngoma et al., 2011). The recently identified copy number variation of *pfplasmepsin2* has been associated with piperaquine resistance (Witkowski et al., 2017; Amato et al., 2017).

Malaria is highly endemic in Sierra Leone, with 2.4 million estimated cases in 2016 (WHO, 2017b). In 2004, the Ministry of Health recommended ASAQ and AL as first- and second-line treatment, respectively, for uncomplicated falciparum infection. In 2015, AL became the first-line drug, as ASAQ was used for mass drug administration during the outbreak of Ebola virus disease in the country and was recommended as second-line treatment. Following a WHO recommendation, the country introduced IPTp with SP more than 10 years ago (Ministry of Health, 2009). Furthermore, the National Malaria Control Programme (NMCP) has recently adopted IPTi with SP and is planning to implement the strategy in 10 high-malaria transmission districts (Ministry of Health, 2015). Evidence of resistance to SP from molecular markers will be vital to inform this plan.

The NMCP of Sierra Leone has established sentinel sites throughout the country for routine monitoring of the efficacy of the recommended ACTs. A therapeutic efficacy study conducted in 2011 at four sites revealed that the recommended first- (ASAQ) and second-line (AL) treatments were highly efficacious (Sahr et al., 2013). Five years later, the NMCP conducted a study to assess the efficacy of three ACTs (AL, ASAQ and DHA/PPQ) and prevalences of known molecular markers for antimalarial drug resistance, including markers for SP resistance. The results of that study are reported here.

## 2 Methods

### 2.1 Study area

**Fig. 1:**
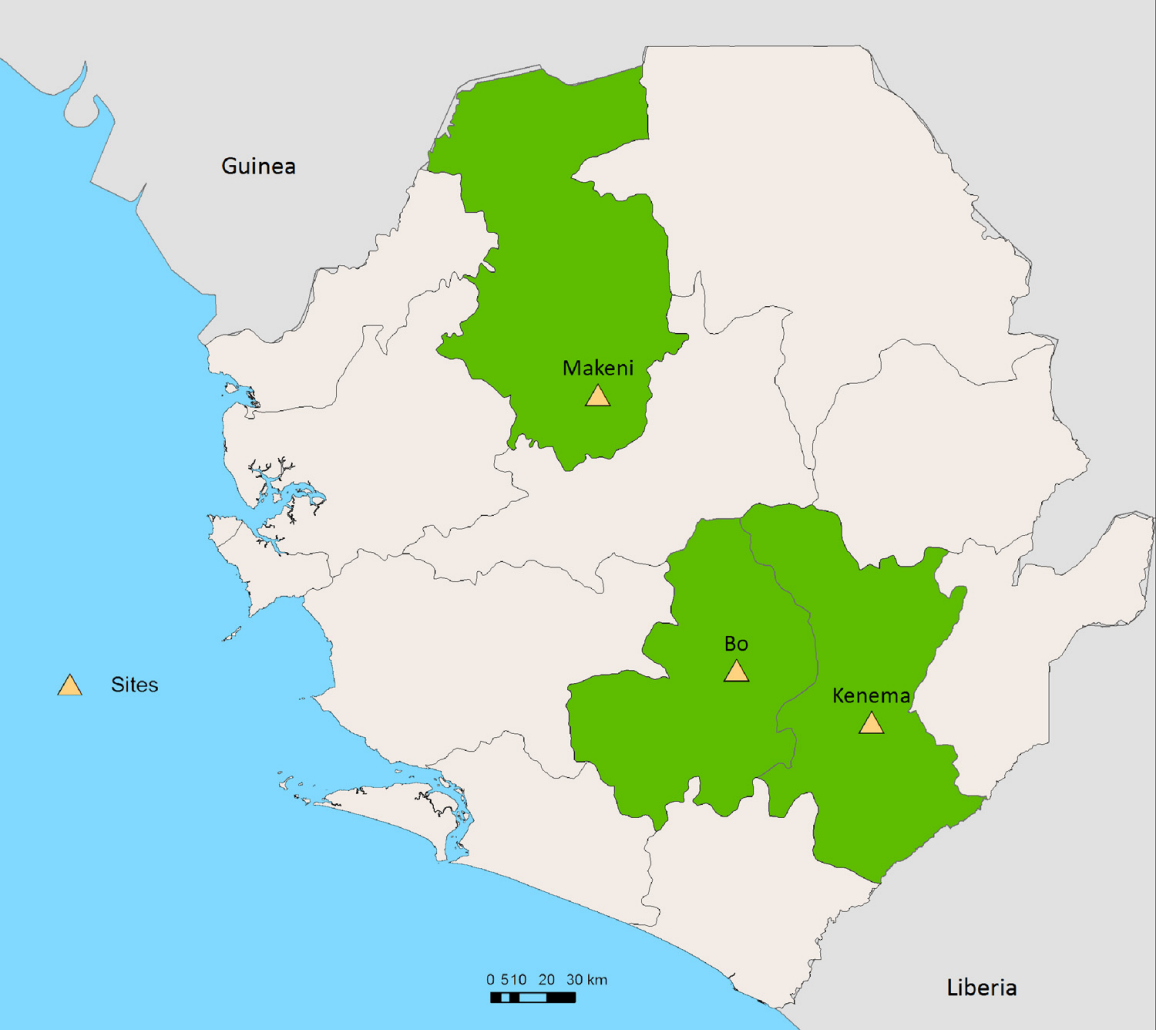
Location of the study sentinel sites.

The study was conducted between March and October 2016 at three sentinel sites Bo, Kenema and Makeni Government hospitals in Bo, Kenema and Bombali districts (Fig. 1). The efficacy of ASAQ was evaluated in Bo and Makeni, AL in Kenema and DHA/PPQ in Bo and Makeni.

### 2.2 Study design and patients

The study was a one-arm prospective assessment of the clinical and parasitological outcomes of directly observed standard therapeutic regimens of ASAQ, AL and DHA/PPQ and the current WHO protocol for assessing the efficacy of antimalarial medicines was used (WHO, 2009). When two different ACTs were assessed at one site, patients were enrolled sequentially; thus, enrolment of patients for the assessment of one ACT was completed before patients were recruited for assessment of the second ACT.

Children aged 6–59 months with an axillary temperature ≥ 37.5 °C or a history of fever during the previous 24 h, *P. falciparum* mono-infection and 500–200 000 asexual parasites/μL of blood were recruited. Patients were not enrolled in the study if they presented with danger signs or severe malaria (WHO, 2013), mixed or mono-infection with *Plasmodium* species other than *P. falciparum*, known hypersensitivity to the study ACTs, severe malnutrition, febrile illness due to non-malaria diseases including measles, acute lower respiratory tract infection, severe diarrhoea with dehydration or other known underlying chronic or severe diseases (*e.g.* cardiac, renal and hepatic diseases, HIV/AIDS). Regular medication that might interfere with the pharmacokinetics of the study ACTs and a history of hypersensitivity reactions to the study medicines were further criteria for exclusion.

### 2.3 Treatment and follow-up

Patients were treated with a daily dose ASAQ or DHA/PPQ for 3 consecutive days or twice daily doses of AL over 3 consecutive days according to the WHO weight-based dose regimen (WHO, 2015). The study medicines were provided by WHO. All treatment doses were given orally, supervised by a designated member of the study team. Patients were observed for 30 min after administration; if they vomited during that period, the full dose was re-administered. If vomiting persisted, the patient was excluded from the study and treated with parenteral artesunate according to the national treatment policy. Patients who failed to respond to the study medicines were treated as follows: those in the ASAQ group received AL, while those in the AL and DHA/PPQ groups received ASAQ.

Day-0 was the day a patient received the first dose of the study medicine. Parents or guardians were invited to bring the study children on scheduled visits on days 1, 2, 3, 7, 14, 21 and 28 for those given ASAQ and AL, continued to days 35 and 42 for those treated with DHA/PPQ, or on an unscheduled day if symptoms occurred. Clinical and laboratory assessments were done during the follow-up visits.

Adverse events, defined as a sign or symptom that was absent at enrolment but manifested during the follow-up period, were assessed by physical examination and by questioning caregivers. Similarly, serious adverse events, defined as conditions that result in death, a life-threatening condition, hospitalization or prolongation of hospitalization, persistent or significant disability or incapacity, were monitored. Both adverse and serious adverse events were recorded on individual case record forms.

### 2.4 Microscope blood examination

Thick and thin blood smears were made during scheduled and unscheduled visits, stained with Giemsa and read independently by two qualified microscopists. The procedures for parasite counting and parasite density calculation were based on the WHO protocol (WHO, 2009). The final parasite density was calculated by averaging the two counts if they were concordant (difference < 50%). If the readings of the two microscopists differed by >50% in terms of parasite positivity, species or parasite density, the blood slides were re-examined by a third independent microscopist. Two concordant results for species and positivity and the average of the two closest densities were considered final results.

### 2.5 Treatment outcomes

Treatment responses were classified as per the WHO protocol (WHO, 2009) as treatment failure (early treatment failure-ETF, late clinical failure-LCF or late parasitological failure-LPF) or treatment success (adequate clinical and parasitological response-ACPR) by 28 days for ASAQ and AL and after 42 days for DHA/PPQ. The proportion of patients who had not cleared parasites by day-3 (day3 positivity rate) was also calculated. The criteria for excluding a patient from the study after enrolment were use of antimalarial drugs by a third party, development of concomitant infection, withdrawal of consent or loss to follow-up.

### 2.6 Parasite genotyping

For patient with recurrent parasitaemia on day-7 onwards, filter paper blood sample was taken on the day of recurrence. Genotypes from day-0 and day of recurrence parasites were compared to differentiate recrudescence from new infection. Specimens were labelled, dried and stored in individual plastic bags with desiccants, protected from light, humidity and extreme temperatures and sent to the Institut Pasteur, for analysis.

Each dried blood spot was punched with a sterile puncher and placed in a 96-well plate in numerical order. Samples were lysed overnight in a saponin solution, and then DNA was extracted with Instagen Matrix resin, as described previously (Canier et al., 2013). DNA samples from day-0 and from the day of recurrence were analysed for genotyping of the highly polymorphic regions merozoite surface proteins 1 and 2 (*msp1, msp2*) and *glutamate-rich protein* (*glurp*) loci. The results were classified as recrudescence if the recurrent parasites were of the same parasite strain as those on day-0 or new infection if they presented with different genotyping profiles.

### 2.7 Molecular markers

All day-0 DNA samples were tested for the presence of mutations in the propeller domain in the *pfK13* gene, which are associated with artemisinin resistance, whereby a portion of the *pfK13* gene was amplified in a nested PCR assay (codons 440–680, 720 bp). Amplicons were sent for sequencing, and DNA sequences were analysed to identify specific single nucleotide polymorphisms (SNPs) related to artemisinin resistance (Menard et al., 2016). Briefly, electrophoregrams were visualized and analysed with CEQ2000 genetic analysis system software (Beckman Coulter). The amino acid sequences were compared with the 3D7 wild-type amino acid sequences PF3D7_1343700. The presence of SNPs was confirmed by reading both the forward and the reverse strands. Parasites with mixed alleles were considered mutants. Copy number variation of *pfplasmepsin2* gene (PF3D7_1408000) was determined in the day-0 samples of the DHA/PPQ-treated group, as described previously by Witkowski et al. (2017). The amplification signals of samples were compared with the signal of a standard control containing a known number of *pfplasmepsin2* copies.

DNA extracts of day-0 samples were also analysed for the presence of mutations in the *pfdhfr* and *pfdhps* genes, as described previously by Andriantsoanirina et al. (2009). Amplicons (600 bp for *pfdhfr* and 724 bp for *pfdhps*) were sequenced and DNA sequences were analysed with CEQ2000 genetic analysis system software (Beckman Coulter). The amino acid sequences were compared with the 3D7 wild-type amino acid sequences (PF3D7_0417200 for *pfdhfr* and PF3D7_0810800 for *pfdhps*). The presence of SNPs was confirmed by reading both the forward and the reverse strands. Parasites with mixed alleles were considered mutants.

### 2.8 Sample size, data management and analysis

A minimum sample size of 50 was calculated after assuming 5% treatment failure of the study medicines, a confidence level of 95% and a precision level of 10%. With a loss to follow-up of 10% by day-28 (ASAQ and AL) or day-42 (DHA/PPQ), 55 patients per site and per drug were required.

All clinical and laboratory data were recorded on standardized case record forms for each patient, and data were validated. Demographic, clinical and laboratory data were double-entered independently and analysed in an Excel® database specifically designed by WHO for studies of antimalarial drug efficacy (http://www.who.int/malaria/publications/atoz/9789241597531/en/).

### 2.9 Ethical considerations

The study protocol was approved by the Sierra Leone Ethics and Scientific Review Committee. Written informed consent was obtained from the parents or guardians of the study children. If the caregiver was illiterate, an accompanying relative or friend served as a witness. Free health care was provided to the children during the study period for any illness related to malaria; this included any expenses related to hospital admission or for treatment of adverse reactions to the medicine. Travel costs for scheduled and unscheduled visits were reimbursed. Patient information was kept confidential.

## 3 Results

### 3.1 Baseline characteristics

Between March and September 2016, 295 (128 for ASAQ, 64 for AL and 103 for DHA/PPQ) eligible children were recruited at the three sites. The baseline profiles of the enrolled patients given the different antimalarial medicines at each site are shown in Table 1. Nine children (six on ASAQ and three on DHA/PPQ) in Bo had an initial parasitaemia above the upper threshold of parasite density (>200 000 asexual parasites/μL).

**Table 1:** Baseline profile of children in a therapeutic efficacy study in three sites, Sierra Leone, 2016.

Characteristic	ASAQ		AL	DP	
	Bo (n = 65)	Makeni (n = 63)	Kenema (n = 64)	Bo (n = 52)	Makeni (n = 51)
Male, n (%)	35(53.8)	33(52.4)	42(65.6)	30(57.7)	24(47.1)
Age (years)					
Mean (SD)	2.5(1.2)	2.4 (1.3)	3.4(1.2)	2.7(1.1)	2.5(1.2)
Range	0.5–4.9	0.5–4.8	0.5–4.9	0.6–4.5	0.6–4.8
Temperature (°C) on day 0					
Mean (SD)	37.3 (0.8)	38.8 (1)	38.1(1)	37.2(0.8)	38.5(0.7)
Parasitaemia (per μL) on day 0					
Geometric mean	25 088	22 032	14 272	21 539	37 230
Range	526–341 625	530–161 857	612–144 533	575–314 739	638–200 000

SD: standard deviation, ASAQ: artesunate+amodiaquine, AL: artemether+lumefantrine, DP: dihydroartemisinin+piperaquine.

### 3.2 Treatment outcomes

Table 2 summarizes the responses to the study medicines at the study sites before and after PCR adjustment. Across all sites, 19 patients were either lost to follow-up or withdrawn from the study. Genotyping indicated that all patients with recurrent infection (n = 30) carried a new infection (n = 25) or their filter paper blood spots were missing and were thus classified as “unknown” (n = 5). PCR-corrected, 100% ACPR was observed among study patients (Table 2). All patients had cleared their parasites by day-3. Patients with an initial parasitaemia > 200 000 asexual parasites/μl either achieved ACPR (n = 6) or were re-infected on day-28, and the treatment outcome was therefore not negatively influenced.

**Table 2:** Treatment responses of children with uncomplicated *P. falciparum* malaria treated with artesunate+amodiaquine (ASAQ), artemether-lumefantrine (AL) or dihydroartemisinin+piperaquine (DP) in three sites, Sierra Leone, 2016.

Outcome	ASAQ (28 days)				AL (28 days)		DP (42 days)			
	Bo (n = 65)		Makeni (n = 63)		Kenema (n = 64)		Bo (n = 52)		Makeni (n = 51)	
	%	95% CI	%	95% CI	%	95% CI	%	95% CI	%	95% CI
*PCR-unadjusted*										
LCF	3(4.9)	1.0–13.7	1(1.6)	0.0–8.7	0(0.0)	0.0–6.4	0(0.0)	0.0–7.5	1(2.0)	0.1–10.6
LPF	8(13.1)	5.8–24.2	3(4.8)	1.0–13.5	5(8.9)	3.0–19.6	5(10.6)	3.5–23.1	4(8.0)	2.2–19.2
ACPR	50(82.0)	70.0–90.6	58(93.5)	84.3–98.2	51(91.1)	80.4–97.0	42(89.4)	76.9–96.5	45(90.0)	78.2–96.7
Total per protocol	61		62		56		47		50	
Withdrawn/lost [n(%)]	4(6.2)		1(1.6)		8(12.5)		5(9.6)		1(2.0)	
Kaplan Meier: cure rate	50(82.3)	70.3–89.8	58(93.5)	83.7–97.5	51(91.1)	79.9–96.2	42(89.4)	76.4–95.4)	45(90.0)	77.6–95.7

*PCR-adjusted*										
LCF	0 (0.0)	0.0–71	0(0.0)	0.0–6.2	0(0.0)	0.0–7.0	0(0.0)	0.0–8.4	0(0.0)	0.0–7.9
LPF	0 (0.0)	0.0–71	0(0.0)	0.0–6.2	0(0.0)	0.0–7.0	0(0.0)	0.0–8.4	0(0.0)	0.0–7.9
ACPR	50(100)	92.9–100	58(100)	93.8–100	51(100)	93.0–100	42(100)	91.6–100	45(100)	92.1–100
Total per protocol	50		58			51	42		45	
Withdrawn/lost	4(6.2)		1(1.6)			8(12.5)	5(9.6)		1(2.0)	
Re-infection/unknown PCR	11(16.9)		4(6.6)			5(7.8)	5(9.6)		5(10)	
Kaplan Meier: cure rate	50(100)		58(100)			51(100)	42(100)		45(100)	

### 3.3 Molecular makers

The day-0 samples of two of the 295 patients were lost. Of the 293 day-0 samples tested for the presence of *pfk13* mutations, 15 did not give interpretable data due to too little or poor-quality DNA. Two hundred and seventy-eight (94.2%) samples gave interpretable sequences. Of these, majority (n = 270; 97.1%) carried wild-type *pfk13* gene and only six had non-synonymous *pfk13* mutant: A578S (n = 3; 1.1%) and I646 T (n = 3; 1.1%). The remaining two were with synonymous *pfk13* mutation: Y493Y (n = 1; 0.4%) and V510 V (n = 1; 0.4%). Among the 103 day-0 samples tested for *pfplasmepsin2* amplification, 100 (97.0%) gave interpretable data, and all (100%) had *pfplasmepsin2* single copy.

**Fig. 2:**
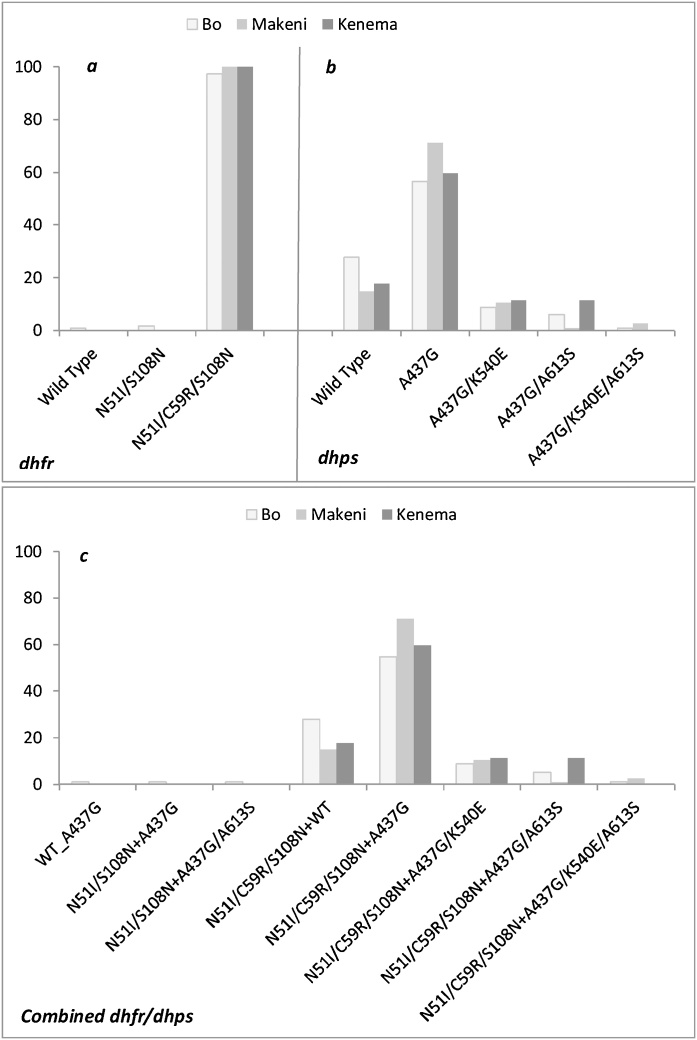
Prevalence of dhfr (**a**), dhps (**b**) and combined dhfr/dhps (**c**) mutations on day-0 samples from children in the study sites in Sierra Leone, 2016.

Of the available 293 samples, 100% and 99.3% (n = 291) gave interpretable data for *pfdhfr* and *pfdhps,* respectively. Fig. 2 presents the proportions of mutations in *dpfhfr*, *pfdhps* separately and combined. All patients in Makeni and Kenema, and 97.4% (n = 112) of patients in Bo carried parasites with a triple *pfdhfr* mutant. The most prevalent *pfdhps* mutation was A437G, accounting for 62.9% (56.5–71.1%) at the three sites, with the highest prevalence in Makeni. The double *pfdhps* A437G/K540E mutant occurred at a prevalence of 10% (8.7–11.3%), and all were part of the quintuple or sixtuple. Combined *pfdhfr/pfdhps* mutation was analysable for 291 patients of which 54.8%–71.1% of patients carried quadruple (N51I/C59R/S108N+A437G) mutant. The overall prevalence of the quintuple (N51I/C59R/S108N+A437G/K540E) mutant was 10%, with no difference among sites: *X^2^* (2, N = 291) = 0.37, *p =0.83*. Another quintuple mutant (N51I/C59R/S108N+A437G/A613S) was found in 14 cases, most of them in Kenema (11.3%) and Bo (5.2%), and a sextuple mutant (N51I/C59R/S108N+A437G/K540E/A613S) in four cases.

## 4 Discussion

Our findings confirm a high cure rate and efficacy (100% ACPR) of the nationally recommended ACTs (ASAQ and AL) for the treatment of uncomplicated falciparum infection in the study areas since their introduction almost a decade ago. A similar response was obtained for DHA/PPQ. Similar results for ASAQ and AL were observed in 2011 at the same sites, indicating that these ACTs have maintained their efficacy. The high cure rate of ASAQ and AL, particularly in children under five years, is encouraging, as treatment failure manifests easily in this age group because of low immunity (WHO, 2010b).

ASAQ and AL are the most commonly recommended ACTs for the treatment of uncomplicated falciparum malaria in African countries. Recent evidence suggests that these ACTs have maintained high efficacy (cure rate ≥ 95%) in many of these countries, despite their use for more than a decade (Yeka et al., 2014; de Wit et al., 2016; Dorkenoo et al., 2016; Ogouyèmi-Hounto et al., 2016; Nega et al., 2016; Paczkowski et al., 2016; Ursing et al., 2016; Sow et al., 2016; Abuaku et al., 2017). However, a lower efficacy (<90% cure rate) of AL was reported from one site in Angola in two consecutive studies in 2013 and 2015 (Plucinski et al., 2015, 2017). Administration of the evening doses of AL in these studies was not supervised, and there was no confirmation that they were given to the patients. Therefore, it is possible that the decreased cure rate was due to sub-therapeutic doses of the treatment; however, the low cure rate observed at only one site in both years might signal consistently reduced AL efficacy in the area. A cure rate below the threshold of 90% calls for a change in treatment policy (WHO, 2010b). Before such action is taken, a confirmatory study of therapeutic efficacy with supervised treatment is urgently needed.

African countries are increasingly adopting DHA/PPQ as second-line treatment. The high cure rate with this ACT observed in our study is in line with other findings from Africa (Ogutu et al., 2014; Ursing et al., 2016; Sow et al., 2016; Plucinski et al., 2017) and might encourage adoption of this ACT as second-line treatment for uncomplicated malaria. The situation is, however, quite different in South East Asia, as treatment failure with DHA/PPQ has recently been reported in Cambodia and Viet Nam, where the combination is being used as first-line treatment. A treatment failure rate with DHA/PPQ of up to 46% was observed in Cambodia in 2012–2013 (Amaratunga et al., 2016), which was associated with a high prevalence of *pfk13* mutations and a high piperaquine 50% inhibitory concentration. In Viet Nam, the rate of treatment failure rate with DHA/PPQ increased from 0 in 2012–2013 to 26% in 2015, with a background of increased molecular markers for artemisinin and piperaquine resistance (Thanh et al., 2017). This was confirmed by Phuc et al. (2017). In addition, Imwong et al. (2017) recently reported the presence of multidrug-resistant parasites carrying both *pfk13* gene mutations and copy number of *pfplasmepsin2* in Cambodia, Thailand and the Lao People's Democratic Republic. The speed with which piperaquine resistance has emerged and consequently compromised the efficacy of DHA/PPQ in the Greater Mekong sub-region is of great concern. African countries that have/are adopting DHA/PPQ as second-line treatment for uncomplicated malaria and those using it for mass drug administration should, therefore, be vigilant in order to detect the emergence of piperaquine resistance.

We observed very low-frequency (2.2%) *pfk13* nonsynonymous mutations, none of which was associated with artemisinin resistance, as assessed by the absence of parasites on day-3. In addition, none is among the known *pfk13* alleles (candidate or confirmed) associated with artemisinin resistance (WHO, 2017a). The A578S mutation was found in three samples. This mutation has commonly been observed in Africa and was recently confirmed as not being associated with artemisinin resistance *in vitro* or *in vivo* (Menard et al., 2016). The absence of *pfk13* mutations associated with artemisinin resistance in this study in Sierra Leone as well as in other African countries (Menard et al., 2016) might suggest that artemisinin resistance has not yet emerged on the continent or spread from the Greater Mekong subregion. The recent emergence and spread of multidrug resistance in the Greater Mekong subregion, however, indicates the importance of continuous monitoring of the efficacy of ACTs and of the molecular markers for resistance to artemisinin and partner drugs. We did not observe *pfplasmepsin2* amplification in the tested samples, suggesting the absence of piperaquine resistance at two sites. This is further confirmed by the ACPR of all patients treated with DHA/PPQ. As this DHA/PPQ is not a nationally recommended ACT, PPQ pressure is unlikely, especially in these remote study areas. However, recent report of piperaquine resistance marker from Mozambique (Gupta et al., 2018), though very low frequency, may underline the need to monitoring appearance of *pfplasmepsin2* amplification particularly in countries recommending the use of DHA/PPQ.

The prevalence of the triple *pfdhfr* (N51I/C59R/S108N) mutant in the study areas was close to or reached fixation, and that of the quadruple (N51I/C59R/S108N+A437G) mutant was >50%; however, that of the quintuple (N51I/C59R/S108N+A437G/K540E) mutant, which is associated with a high level of SP clinical failure (Omar et al., 2001; Kublin et al., 2002; Staedke et al., 2004), was low. These findings are in line with previous reports from West Africa, where the triple *pfdhfr* mutant is common but the *pfdhps* K540 and consequently the quintuple mutants are rare or absent (Ruizendaal et al., 2017; Okell et al., 2017). In contrast, the quintuple mutant is highly prevalent in East Africa, approaching or reaching 100% (Okell et al., 2017). Furthermore, recent evidence of parasites carrying an additional *pfdhps* mutation at codon A581G (Okell et al., 2017) together with the quintuple (N51I/C59R/S108N+A437G/K540E) mutant suggests intensification of SP resistance. This sextuple was found to be associated with high clinical failure and loss of protective efficacy of IPTi with SP (Gosling et al., 2009) and IPTp (Minja et al., 2013; Gutman et al., 2015; Chico et al., 2015; Okell et al., 2017). WHO recommends IPTi with SP only in areas of high-to-moderate malaria transmission, where the prevalence of K540E among infected individuals is <50% (WHO, 2010c). Therefore, the very low prevalence of the quintuple mutant in the current study in Sierra Leone supports the NMCP’s decision to introduce IPTi with SP in high-malaria transmission districts in order to curb the incidence of malaria and its complication in infants.

## 5 Conclusion

ASAQ, AL and DHA/PPQ were found to be highly effective for the treatment of uncomplicated malaria in the study areas, and neither *pfk13* mutations (associated with artemisinin resistance) nor *pfplasmepsin2* multiple copies (associated with piperaquine resistance) were found. Furthermore, the very low prevalence of the quintuple mutant in the current study supports the NMCP’s decision to introduce IPTi with SP in selected high-malaria transmission districts.

## Authors’ contributions

SJS: conceived and designed the study, supervised the field work and contributed to writing the manuscript.

ARYK: conceived and designed the study, conducted the field work and contributed to writing the manuscript.

FS: contributed to the study design, study conduct and preparation of the manuscript

MS: contributed to the study design, study conduct and preparation of the manuscript

ASS: participated in the field work, responsible for the quality assurance and quality control of the malaria microscopy and participated in the writing of the manuscript.

DM: responsible for the analysis of parasite genotyping and molecular markers for artemisinin and piperaquine resistance and contributed to the write up of the manuscript.

MW: contributed to the study design, validated and analysed data and contributed to preparation of the manuscript

All the authors read and approved the final version of the manuscript.

## Disclaimer

Marian Warsame is a staff member of the World Health Organization, and she alone is responsible for the views expressed in this publication, which do not necessarily represent the decisions, policy or views of the World Health Organization.

## Competing interests

The authors declare no competing interests.

**Trial registration number: ACTRN12618000517279**.
